# Role of vitamin D supplementation in the management of musculoskeletal diseases: update from an European Society of Clinical and Economical Aspects of Osteoporosis, Osteoarthritis and Musculoskeletal Diseases (ESCEO) working group

**DOI:** 10.1007/s40520-022-02279-6

**Published:** 2022-10-26

**Authors:** Thierry Chevalley, Maria Luisa Brandi, Kevin D. Cashman, Etienne Cavalier, Nicholas C. Harvey, Stefania Maggi, Cyrus Cooper, Nasser Al-Daghri, Oliver Bock, Olivier Bruyère, Mario Miguel Rosa, Bernard Cortet, Alfonso J. Cruz-Jentoft, Antonio Cherubini, Bess Dawson-Hughes, Roger Fielding, Nicholas Fuggle, Philippe Halbout, John A. Kanis, Jean-Marc Kaufman, Olivier Lamy, Andrea Laslop, Maria Concepción Prieto Yerro, Régis Radermecker, Jotheeswaran Amuthavalli Thiyagarajan, Thierry Thomas, Nicola Veronese, Marten de Wit, Jean-Yves Reginster, René Rizzoli

**Affiliations:** 1 Service of Bone Diseases, Geneva University Hospitals and Faculty of Medicine, Geneva, Switzerland; 2 Metabolic Bone Diseases Unit, Department of Surgery and Translational Medicine, University of Florence, Florence, Italy; 3 Cork Centre for Vitamin D and Nutrition Research, School of Food and Nutritional Sciences, University College Cork, Cork, Ireland; 4 Department of Clinical Chemistry, University of Liege, CHU de Liege, Liege, Belgium; 5 MRC Lifecourse Epidemiology Centre, University of Southampton, Southampton, UK; 6 NIHR Southampton Biomedical Research Centre, University of Southampton and University Hospital Southampton NHS Foundation Trust, Southampton, UK; 7 CNR Aging Branch-IN, Padua, Italy; 8 UKNIHR Oxford Biomedical Research Centre, University of Oxford, Oxford, UK; 9 Chair for Biomarkers of Chronic Diseases, Biochemistry Department, College of Science King Saud University, Riyadh, 11451 Saudi Arabia; 10 Department of Osteoporosis, Inselspital, Bern University Hospital, University of Bern, Bern, Switzerland; 11 International Osteoporosis Foundation, Nyon, Switzerland; 12 Division of Public Health, Epidemiology and Health Economics, WHO Collaborating Center for Public Health Aspects of Musculo-Skeletal Health and Ageing, University of Liège, Liege, Belgium; 13 Centro de Estudos Egas Moniz Faculdade de Medicina da Universidade de Lisboa, Lisbon, Portugal; 14 Department of Rheumatology, University of Lille, CHU Lille, MABlab ULR 4490, Lille, France; 15 Servicio de Geriatría, Hospital Universitario Ramón y Cajal (IRYCIS), Madrid, Spain; 16 Dipartimento dei percorsi geriatrici della fragilità, Geriatria, Accettazione geriatrica e Centro di ricerca per l’invecchiamentodella continuità di cura e riabilitativi, IRCCS INRCA, Ancona, Italy; 17 Jean Mayer USDA Human Nutrition Research Center on Aging at Tufts University, Boston, MA USA; 18 Centre for Metabolic Bone Diseases, University of Sheffield Medical School, Sheffield, UK; 19 Mary McKillop Institute for Health Research, Australian Catholic University, Melbourne, Australia; 20 Department of Endocrinology, Ghent University Hospital, Ghent, Belgium; 21 Bone Unit, Lausanne University Hospital and University of Lausanne, Lausanne, Switzerland; 22 Scientific Office, Federal Office for Safety in Health Care, Austrian Medicines and Medical Devices Agency, Vienna, Austria; 23 Spanish Agency for Medicines and Medical Devices, Madrid, Spain; 24 Department of Clinical Pharmacology Diabetes, Nutrition and Metabolic Disorders, CHU Liege, Liège, Belgium; 25 Ageing and Health Unit, Department of Maternal, Newborn, Child and Adolescent Health & Ageing, WHO HQ, Geneva, Switzerland; 26 Department of Rheumatology, North Hospital, CHU Saint-Etienne and INSERM U1059, University of Lyon-University Jean Monnet, Saint-Etienne, France; 27 Geriatric Unit, Department of Internal Medicine and Geriatrics, University of Palermo, Palermo, Italy; 28 Department of Medical Humanities, Amsterdam University Medical Centre, Amsterdam, The Netherlands; 29 Department of Public Health, Epidemiology and Health Economics, University of Liège, Liège, Belgium

**Keywords:** Vitamin D, Fragility fracture, Falls, Osteoarthritis

## Abstract

Vitamin D is a key component for optimal growth and for calcium–phosphate homeostasis. Skin photosynthesis is the main source of vitamin D. Limited sun exposure and insufficient dietary vitamin D supply justify vitamin D supplementation in certain age groups. In older adults, recommended doses for vitamin D supplementation vary between 200 and 2000 IU/day, to achieve a goal of circulating 25-hydroxyvitamin D (calcifediol) of at least 50 nmol/L. The target level depends on the population being supplemented, the assessed system, and the outcome. Several recent large randomized trials with oral vitamin D regimens varying between 2000 and 100,000 IU/month and mostly conducted in vitamin D-replete and healthy individuals have failed to detect any efficacy of these approaches for the prevention of fracture and falls. Considering the well-recognized major musculoskeletal disorders associated with severe vitamin D deficiency and taking into account a possible biphasic effects of vitamin D on fracture and fall risks, an European Society for Clinical and Economic Aspects of Osteoporosis, Osteoarthritis and Musculoskeletal Diseases (ESCEO) working group convened, carefully reviewed, and analyzed the meta-analyses of randomized controlled trials on the effects of vitamin D on fracture risk, falls or osteoarthritis, and came to the conclusion that 1000 IU daily should be recommended in patients at increased risk of vitamin D deficiency. The group also addressed the identification of patients possibly benefitting from a vitamin D loading dose to achieve early 25-hydroxyvitamin D therapeutic level or from calcifediol administration.

## Introduction

Vitamin D plays a major role in optimal growth and in calcium–phosphate homeostasis. In humans, skin photosynthesis is the main endogenous source of vitamin D. When sun exposure is limited and in case of insufficient vitamin D dietary supply, vitamin D supplementation is recommended particularly in certain age groups [1, 2]. A majority of guidelines consistently recommend 400 IU/day (10 µg) to prevent rickets during the first year of life [3]. Calcium intake of 200 and 260 mg/day before 6 months and before 12 months, respectively, is also advised [1]. For treatment of nutritional rickets, the dose of 2000 IU/day for at least 3 months is recommended, with a normalization of PTH levels. Whilst recommendations for rickets prevention and treatment in childhood and adolescence are widely accepted [3], the target for calcifediol (25-hydroxyvitamin D (25(OH)D)) levels is still discordant among the various guidelines [2]. Regarding the preventive effect of vitamin D on fracture, many meta-analyses of RCTs have shown contradictory results, some showing no effect at all, while others showed a beneficial anti-fracture effect of vitamin D supplementation particularly when combined with calcium [2, 4].

Although 50 nmol/L of serum of 25(OH)D is considered as a threshold value for optimal bone health by many guidelines [3], higher concentrations, and correspondingly higher intake of vitamin D, have been suggested by some experts for maintaining bone health [5–8]. However, recent large trials conducted in vitamin D-replete healthy subjects have reported no effects either on falls [9, 10], non-vertebral fractures [9, 11], bone health outcomes [10], chronic knee pain [12], various clinical outcomes [13], and body composition [14]. It should be noted that 70% of RCTs with clinical outcomes were performed in patients with baseline 25(OH)D above 40 nmol/L and out of twenty-five large RCTs only one was undertaken in a vitamin D-deficient population (25(OH)D below 25 or 30 nmol/L) and two in vitamin D-insufficient populations (25(OH)D below 50 nmol/L) [15]. Indeed, vitamin D deficiency has been shown to be a risk factor for various musculoskeletal disorders, including fractures and falls [16, 17]. To identify vitamin D regimens to correct vitamin D deficiency and prevent related musculoskeletal events, a European Society for Clinical and Economic Aspects of Osteoporosis, Osteoarthritis and Musculoskeletal Diseases (ESCEO) working group reviewed and analyzed meta-analyses of randomized controlled trials on the effects of vitamin D supplementation on fracture risk, falls or osteoarthritis. This paper, which updates the 2013 ESCEO recommendation paper [18], is a narrative review of the data used by the working group to reach the conclusion that 1000 IU vitamin D daily should be recommended in patients at increased risk of vitamin D deficiency.

## Methods

An ESCEO expert working group, comprising expert clinicians of different medical specialties (physical rehabilitation medicine (PRM) specialists, laboratory medicine specialists, rheumatologists, endocrinologists and geriatricians) and researchers was convened in May 2022, under the auspices of the WHO Collaborating Center for Epidemiology of Musculoskeletal Health and Aging (University of Liège, Belgium). This group reviewed the literature and discussed the current state of the art on the followings topics: (1) Epidemiology of vitamin D deficiency; (2) New perspectives in the assessment of vitamin D circulating levels; (3) Vitamin D and fracture prevention (effects in depleted and replete populations); (4) Vitamin D and falls (effects in depleted and replete populations); (5) Vitamin D and osteoarthritis (effects in depleted and replete populations); (6) Metabolic conditions justifying the use of 25(OH)D (calcifediol); and (7) Vitamin D treatment safety and therapeutic window. This paper reflects the presentations and the discussions of the working group based on an extensive narrative literature review focusing on the most robust evidence such as a series of recent meta-analyses*.* After discussion and deliberation amongst the experts regarding the quality, scope, and context of the collected evidence, a first draft of the manuscript was prepared. This draft manuscript was circulated to all the members of the expert group for critical revision. Any additionally identified high-quality evidence was subsequently incorporated. The first author coordinated the preparation of the final version of the manuscript, which was circulated to the entire expert group for final approval and unanimous endorsement.

### Epidemiology of vitamin D deficiency

Vitamin D deficiency varies depending on the thresholds used for serum 25(OH)D values [19]. Furthermore, serum 25(OH)D levels vary over the course of the year in relation to UVB exposure [20–22]. Low thresholds such as 25(OH)D below 25 or 30 nmol/L (i.e., deficiency or severe deficiency, depending on the experts society) have been suggested when the focus was more on the prevention of rickets/osteomalacia and a higher threshold below 50 nmol/L (insufficiency or deficiency, depending on the experts society) was chosen if the concern was suppression of PTH. Using a < 30 nmol/L threshold, 5% of Americans and about 9% of Canadians have serum values of 25(OH)D below this level [22, 23]. Regarding vitamin D status in the United States, an American survey between 2011 and 2014 suggested that, on average, 5% of the population was at risk of deficiency as defined with 25(OH)D level below 30 nmol/L, but if the population was divided according to the ethnicity, then the risk of vitamin D deficiency of the Caucasian population was around 2% as compared to 17.5% of the black population [23]. In Europe, 1 out of 8 inhabitants (13% on average) are at risk of vitamin D deficiency (based on a 25(OH)D level below 30 nmol/L), but this percentage can increase up to 28–65% for ethnic minority groups within Northern European countries [24]. However, the prevalence of serum 25(OH)D below 30 nmol/L ranged from 4.6 to 30.7% and this range increased from 27.2 to 61.4% for 25(OH)D using a threshold of 50 nmol/L according to European standardized data [24]. Considering both continents, this represents 120 million of subjects who are vitamin D deficient using a 30 nmol/L threshold and 390 million of subjects with 25(OH)D below 50 nmol/L. Predictors for low 25(OH)D in all models were higher age, higher BMI, use of walking aid, limited time spent outdoors in Summer, smoking, no calcium supplementation, no use of multivitamins, no use of vitamin D on prescription or self-administered, and the season when serum 25(OH)D was tested [25]. There are other potential factors that contribute to increased risk of vitamin D deficiency such as inflammation [5, 26, 27], patients with fat malabsorption syndromes, bariatric surgery, nephrotic syndrome, as well as patients on a variety of medications which may interfere with vitamin D absorption or metabolism [5].

The major source of vitamin D in humans is ultraviolet B (UVB)-induced dermal synthesis of cholecalciferol, whereas food sources are believed to play a lesser role, although among white residents of the UK, meat eaters have 20 nmol/L higher 25(OH)D than vegans [28]. There is a clear trend of decreasing UVB exposure by moving from South to North within Europe, with an almost six-fold difference in mean yearly modeled UVB dose between these two latitudes from ~ 35^○^ to ~ 69^○^ N [29]. Significant differences in UVB availability between inland and coastal areas were also reported at the same latitude within the UK, due to different cloud conditions [30]. Environmental and personal factors influence cutaneous vitamin D synthesis [31]: the main determinants are solar zenith angle, controlling the potential available UVB (latitude, season and time of day), skin type and age, clothing, sunscreen, and outdoor activities**.** Therefore, UVB availability and diet, as well as other lifestyle factors, must be considered in preventive and therapeutic approaches to vitamin D deficiency. In Europe, if the extreme North and South are excluded, dietary supply becomes especially important 4–8 months a year when vitamin D synthesis in the skin is very limited and 50–100% of Europeans have vitamin D intakes below the recommended level. Indeed, the vast majority of European countries consume below the estimated average requirement of 10 ug/day of vitamin D intake (400 IU) based on European dietary surveys with a mean intake of 3.3 and 2.7 ug/day in adult males and females, respectively [32]. The mean vitamin D intake varies between 1.5 and 5 μg/day in Western Europe and below 1 μg/day to about 3 μg/day in Southern Europe, while in Northern Europe it ranges between 4 and 14 μg/day [33]. According to WHO, the strategies for the control of micronutrient malnutrition are to increase the diversity of the foods consumed, food fortification and supplementation [34]. Nevertheless, increasing the diversity of foods consumed and improving intake of naturally occurring vitamin D‑rich foods is particularly challenging because there are very few food sources rich in vitamin D. For a serum 25(OH)D threshold of 25 nmol/L, a vitamin D intake range of 7.5 and 10 µg/day would allow 95% and 97.5% of individuals, respectively, to maintain their winter serum 25(OH)D above this threshold. On the other hand, for a serum 25(OH)D threshold of 50 nmol/L, the corresponding vitamin D intakes needed are 23.5 and 25 ug/day (≈ 1000 IU/day), respectively, intakes which are too difficult to reach with food fortification and may need a more active public health approach involving recommendations for lifestyle including sunshine exposure, healthy nutrition, food fortification, and vitamin D supplementation [35].

In summary, vitamin D deficiency (25(OH)D below 25 or 30 nmol/L) is evident throughout the European population at prevalence rates that are of concern and that require action both from a public health and a clinical perspective.

### New perspectives in the assessment of vitamin D circulating levels

Commonly cited indications where vitamin D testing is appropriate are: rickets, osteomalacia, osteoporosis, hyperparathyroidism, malabsorption syndromes, medications affecting absorption or metabolism of vitamin D (antifungals, HIV antiretroviral therapy, anticonvulsants, etc.), chronic kidney disease, hypophosphatemia and hypo/hypercalcemia, deeply pigmented skin, and isolated elevation of alkaline phosphatase [36]. A major issue in the determination of a 25(OH)D threshold value is the absolute need for a standardized method. In 2010, the Vitamin D External Quality Assessment Scheme (DEQAS) for the determination of 25(OH)D included 13 different methods which displayed high coefficient of variation and a range of fourfold between the lowest and highest values for a given sample. To standardize 25(OH)D measurement in both clinical and research laboratories, the Vitamin D Standardization Program (VDSP) was agreed in 2010 by the Office of Dietary Supplements (ODS) of the National Institutes of Health (NIH) [37]. This collaboration involved the coordinated efforts of ODS, the National Institute for Standards and Technology (NIST), the Centers for Disease Control and Prevention (CDC), the Vitamin D External Quality Assessment Scheme (DEQAS), the College of American Pathologists (CAP), the American Association for Clinical Chemistry (AACC), the International Federation of Clinical Chemistry and Laboratory Medicine (IFCC), along with national surveys and collaborators around the world. The VDSCP calibration-certification process conducted by CDC comprised 2 phases: Phase 1 assessed and readjusted current calibration based on 40 single donor samples with assigned target values. In Phase 2, four quarterly challenges with 10 unknown serum samples were performed to verify that calibration was stable over 1 year. If performance criteria of a mean bias ≤  ± 5% and an imprecision ≤ 10% were reached, then the certified labs were listed on the CDC website https://www.cdc.gov/labstandards/pdf/hs/CDC_Certified_Vitamin_D_Procedures-508.pdf. In 2018, the DEQAS reported an explosion of the number of methods for 25(OH)D determination, but with lower coefficient of variation values as compared to 2010 and a narrowing distribution with a smaller range of twofold between the lowest and highest values for a given sample. The impact of the VDSP re-standardization was an overall improvement in the bias observed between the reference method and the different commercially available assays. Liquid chromatography–mass spectrometry (LCMS/MS) methods performed globally better than immunoassays, but all LCMS/MS methods are not equivalent and there are still important issues with immunoassays and LCMS/MS.

All the standardization process was performed with samples from healthy individuals, but 25(OH)D should also be measured in patients with different diseases (CKD, osteoporosis, etc.), physiological status (pregnancy), or ethnicity. Vitamin D binding protein concentration and polymorphism have also an impact on the results. In addition serum matrix may be different in sick patients and in healthy individuals [38].

The vitamin D metabolite ratio (VMR) 24,25(OH)_2_D to 25(OH)D has been proposed as a new marker of vitamin D status and as being predictive of fracture risk [39]. Cavalier et al. suggested that in clinical practice the concentrations of 24,25(OH)_2_D and 25(OH)D should be reported together with the probability that a ratio value is found in healthy subjects [40]. This information would allow one to escape the concept of vitamin D deficiency solely on the basis of a 25(OH)D threshold [5, 41–43]. For example, amongst individuals with a 25(OH)D concentration above 52 nmol/L, over 99% exhibit detectable amounts of 24,25(OH)_2_D and, thus, are probably vitamin D sufficient [44]. This finding supports the 50 nmol/L cut-off recommended by the IOF [45]. However, the ratio 24,25(OH)_2_D to 25(OH)D can only be measured after determination of both moieties by LCMS/MS.

In summary, great improvements in 25(OH)D assays standardization have been achieved in recent years. LCMS/MS methods generally perform better than immunoassays, but all LCMS/MS methods are not equivalent. 24,25(OH)2D and VMR are promising tools to evaluate vitamin D deficiency at the individual level, but are only available with LCMS/MS.

### Vitamin D and fracture risk

In a prospective observational study in 1662 community-dwelling elderly men followed over a period of 4 years, a U-shaped association was observed with an increased risk of fracture in men with either 25(OH)D values ​​below 36 nmol/L or above 75 nmol/L [46]. Whether vitamin D, calcium or both supplements together are effective for the primary prevention of fractures is still debated. The absence or the demonstration of a beneficial anti-fracture effect is linked not only to the vitamin D dose, addition or not of calcium and duration of vitamin D supplementation but also largely to the population studied and in particular if this population is vitamin D deplete or replete.

In a Cochrane review [47], taking into account 11 studies involving more than 27,000 patients, vitamin D alone did not prevent hip fractures or any fractures (the latter based on 15 studies including more than 28,000 patients) (Table 1). On the other hand, the combination of vitamin D and calcium reduced the incidence of hip fractures by 16% based on 9 studies in almost 50,000 patients and the risk of any fractures by 5% taking into account 10 studies involving nearly 50,000 patients. In addition, fracture risk reduction was shown to be more important in institutionalized patients both for hip and for any fractures [47]. Nevertheless, even in institutionalized patients, the reduction of fracture incidence seems to be dependent on baseline serum 25(OH)D levels. Indeed, in 3,270 French institutionalized women with a mean age of 85 years and a baseline serum 25(OH)D of 35 nmol/L, a supplementation of 800 IU of vitamin D and 1200 mg of calcium for 18 months reduced the incidence of hip and of non-vertebral fractures [48]. In contrast, in an English study among 3440 institutionalized men and women with a mean age of 85 years, but with a baseline 25(OH)D above 54 nmol/L, a supplementation of 100,000 units of vitamin D every 4 months for 3 years did not show a significant decrease in the incidence of fractures at the hip or at other sites [49]. More recently the Do-Health study involving 2157 elderly subjects with a baseline 25(OH)D of 56 nmol/L, a daily supplementation of 2000 IU of vitamin D for 3 years did not reduce the incidence of non-vertebral fractures [13]. In the VITAL trial performed in more than 25,000 vitamin D-replete subjects, there was no anti-fracture effect of 2000 IU/day of vitamin D [11]. With only 7 against 8 fractures in patients with 25(OH)D below a not prespecified 30 nmol/L threshold, a conclusion as to whether a response to supplementation is detectable in vitamin D-deficient patients cannot be drawn.

**Table 1:** Meta-analyses on vitamin D supplementation and fractures

Author, year, studies	Intervention tested	Target population and limitations	Results for fractures
Bischoff-Ferrari et al. (2009) [51] 12 RCTs (non- vertebral fractures) *N* = 42,279 8 RCTs (hip fractures) *N* = 40,886	**Vit D ± calcium** Vit D ≤ 400 IU/d in 3 trials whereas the other 9 RCTs had mean intakes of 482–770 IU/d	Mean age of 78 years, 89% women Duration from 12 to 84 months	**Pooled relative risk 0.86 (95% CI 0.77–0.96) for prevention of non-vertebral fracture and 0.91 (95% CI 0.78–1.05) for hip fracture**
	Calcium 500–1200 mg/d in combination with vit D in 7 RCTs		**For the higher dose (> 400 IU), pooled RR 0.80 (95% CI 0.72–0.89); *n* = 33,265, 9 trials for non-vertebral fractures and 0.82 (95% CI 0.69–0.97); *n* = 31,872, 5 trials for hip fractures**
Lai et al. (2010) [52] 7 RCTs *N* = 12,762	**Vit D alone** 400–1100 IU/day in 6 RCTs Vit D 800 IU + calcium 1000 mg/day in 1 RCT	RCT including a minimum of 100 participants with at least one radiologically confirmed hip fracture in each group	RR 1.13 (95% CI 0.98–1.29) for hip fractures No significant variations found between results of studies randomizing participants to: < 800 IU/day 1.14 (95% CI 0.86–1.49) or ≥ 800 IU/day 1.12 (95% CI 0.96–1.32)
Bergman et al. (2010) [53] 8 RCTs *N* = 12,658	**Vit D ± calcium**	4 RCTs on non-vertebral fracture, *n* = 3510 5 RCTs on hip fracture, *n* = 7473	**Vit D3 + calcium compared to placebo:** **OR 0.77 (95% CI 0.6–0.93) for non-vertebral fracture and OR 0.70 (95% CI 0.53–0.90) for hip fracture**
Chung et al. (2011) [54] 16 RCTs *N* = 19,878	**Vit D (400–1370 IU/d) alone** 5 RCTs—*N* = 14,583	Elderly men and women with follow-up ranging from 7 months to 5 years	Pooled relative risk 1.03 [95% CI 0.84 to 1.26], with high heterogeneity
	**Vit D (300–1000 IU/d) + calcium** 500–1200 mg/d 11 RCTs—N = 52,915		**Pooled relative risk, 0.88 [95% CI 0.78–0.99], with moderate heterogeneity** **Significant risk reduction among institutionalized elderly persons (relative risk, 0.71 [95% CI 0.57–0.89])**
Murad et al. (2012) [55] Network meta-analysis	**Vit D ± calcium**	Median age, 64 years; 86% females and 88% Caucasians; median follow-up, 24 months	**OR 0.81 (95% CI 0.68–0.96) for hip fracture** and OR 0.94 (95% CI 0.84–1.02) for non-vertebral fracture Vit D and calcium given separately were ineffective
Avenell et al. (2014) [47] 31 RCTs *N* = 36,282	**Vit D alone** 11 RCTs, N = 27 693 15 RCTs, N = 28 272	12/31 RCTs had participants with a mean or median age of 80 years or over	RR 1.12 (95% CI 0.98–1.29) for hip fracture RR 1.03 (95% CI 0.96–1.11) for any fracture
	**Vit D + calcium** 9 RCTs, *N* = 49 853 10 RCTs, *N* = 49 976 *Institutional (2 RCTs), N* = *3853*	Prevention of fractures in community, nursing home or hospital inpatient populations	**RR 0.84 (95% CI 0.74–0.96) for hip fracture** **RR 0.95 (95% CI 0.90–0.99) for any fracture** ***RR 0.75 (*95% CI *0.62, 0.92)* and *RR 0.85 (*95% CI *0.74, 0.98) for hip and any fracture, respectively in institutionalized-dwelling subjects***
Weaver et al. (2016) [56] 8 RCTs *N* = 30,970	**Vit D + calcium**	Mostly adults aged 65 + years – Excluded studies that tested vit D without calcium – Included 40% of the literature that contributed to current guidelines on vit D	**15% reduction of total fractures (RR = 0.85; 95%** **CI 0.73–0.98)** **30% reduction of hip fractures (RR = 0.70; 95%** **CI 0.56–0.87)**
Zhao et al. (2017) [57] 33 RCTs (*n* = 51,145)	**Vit D, Calcium and Vit D + calcium**	Community-dwelling participants aged 50 + years for primary prevention without a prior fracture; baseline 25(OH)D ≈ 50 nmol/L Exclusion of older adults living in institutions, most vulnerable to low calcium intake, vitamin D deficiency and fracture risk 11 out of 33 with follow-up of ≤ 12 months with little potential to show benefit on fracture reduction, 4 trials had an open study design without a treatment in the control group No adjustment for adherence For vit D alone 8 of the 12 trials gave vit D in bolus doses (orally or intramuscular administration), which has repeatedly raised concerns in the literature about promoting both falls and fractures	No significant effect of calcium, vit D or both on risk of hip fracture compared with placebo or no treatment: Calcium: RR = 1.53 (95% CI 0.97–2.42) Vit D: RR = 1.21 (95% CI 0.99–1.47) Vit D + calcium RR = 1.09 (95% CI 0.85–1.39) No significant benefit on any intervention on the incidence of non-vertebral, vertebral, or total fractures
Kahwati et al. (2018) [58] 11 RCTs (*N* = 51,419)	**Vit D, Calcium and Vit D + calcium**	Community-dwelling adults aged 50 + years not at risk for osteoporosis or vit D deficiency Panel acknowledged limited trial data for primary prevention	For vit D doses greater than 400 IU (according to current recommendations), the panel concluded that there is insufficient evidence to assess a benefit from vit D
Bolland et al. (2018) [59] 81 unblinded and blinded randomized trials among (*n* = 44,790)	**Vit D compared to untreated controls, placebo or another dose of vit D**	Adults aged 50 + years Authors excluded trials that combined vit D with calcium and thereby 40% of the literature that contributed to current guidelines Authors included large bolus doses that have consistently increased the risk of falls and fractures Biased reporting on low-dose vit D with 800 IU vit D trials	No benefit on fractures **Re-analysis of 800–1000 IU vit D trials of this meta-analysis and excluding bolus trials suggests a significant 14% reduction in total fractures**
Hu et al. (2019) [60] 25 RCTs *N* = 43,510	**Vit D, Calcium and Vit D + calcium**	Adults aged older than 50 years and living in their communities and only studies that lasted more than a year	No reduction of the risk of total, hip and vertebral fractures using different concentrations of vit D, calcium or their combination compared with placebo or no treatment
Yao et al. (2019) [61] 11 RCTs *N* = 34,243 6 RCTs *N* = 49,282	**Vit D alone** (daily or intermittent dose of 400–30 000 IU) 11 RCTs	Mean age 65.9–85.0 years, baseline 25(OH)D 26.5–65.8 nmol/L, mean duration 3 yrs. Yielding a median difference in 25[OH]D concentration of 21 nmol/L	Vit D alone: no reduction of risk of any fracture (RR 1.06; 95% CI 0.98–1.14) or hip fracture (RR 1.14; 95% CI 0.98–1.32)
	**Vit D (400–800 IU daily) + calcium (1000–1200 mg daily)** 6 RCTs	Constrained by infrequent intermittent dosing, low daily doses of vit D, or an inadequate number of participants Yielding a median difference in 25(OH)D concentration of 23 nmol/L	**↓ 6% of any fracture (RR 0.94; 95% CI 0.89–0.99) and** **↓ 16% of hip fracture (RR 0.84; 95% CI 0.72–0.97)**
Eleni et al. (2020) [62] 10 RCTs *N* = 74,325	**Vit D + calcium**	Patients aged 50 years or older Reported on fractures as a primary outcome	**RR 0.74 (95% CI 0.58–0.94) for total fracture** **RR: 0.61 (95% CI 0.4–0.92) for hip fracture** **8 RCTs, *N* = 68,957**
Thanapluetiwong et al. (2020) [63] 26 RCTs *N* = 40,209	**Vit D ± calcium**	Major populations were elderly women with age less than 80 years	Vit D alone failed to show any fracture lowering benefit, RR 0.949 (95% CI 0.846–1.064) **Vit D + calcium significantly lower fracture rates,** **RR 0.859 (95% CI 0.741–0.996)**
Li et al. (2021) [64] 33 RCTs *N* = 83,083	**Vit D ± calcium**	No younger than 47 years old; Follow-up ranged from 3 to 84 months Fracture cases confirmed with hospital diagnosis, medical records, or World Health Organization diagnostic criteria; subjects not treated with any osteoporosis medications and did not have any special physical training;	Vit D alone: no reduction of the risk of total fractures (RR 0.96 (95% CI 0.87–1.05) **Vit D3 (700–800 IU/d) + calcium: significant reduction of total (RR 0.85 (95% CI 0.77–0.95), hip (RR 0.81 (95% CI 0.68–0.97), and non-vertebral fractures (RR 0.84 (95% CI 0.74–0.95), in a pairwise meta-analysis**
Chakhtoura et al. (2022) [65] Umbrella review of meta-Analyses 25 RCTs	**Vit D + calcium—13 SR/MAs**	Mean age between 62 and 85 years	**Vit D + calcium:** **↓ risk of hip fractures in 8/12 SRs/MAs (RR 0.61–0.84)**
	**Vit D alone—19 SR/MAs** Vit D dose 400–800 IU/day	Trials extended from 1 to 7 years 5 SRs/MAs reported on baseline 25(OH)D (20.9–83.8 nmol/L)	**↓ risk of any fractures in 7/11 SRs/MAs (RR 0.74–0.95)**
	6 SR/MAs included 1 trial providing a high dose of 300,000 IU once Calcium dose was 500–1200 mg/day		**No fracture risk reduction in SRs/MAs exclusively evaluating community-dwelling individuals or in those on vit D alone compared to placebo/control**
Kong et al. (2022) [66] 32 RCTs N = 104,363 16 RCTs *N* = *36.793* for fracture outcome	**Vit D (median dose of 800 IU/d) ± calcium** 8 studies reported < 800 IU/day	Most studies included women with 75% of participants (range 15–100%)	**Vit D 800–1000 IU/d:** **Pooled relative risk 0.87 (95% CI 0.78–0.97) for osteoporotic fractures**
	15 studies 800–1000 IU/day, and	Median age was 72 years (range 53–85)	No reduction of hip fractures RR 0.84 (95% CI 0.64–1.10
	9 studies > 1000 IU/day 26 studies reported daily administration, while 6 reported intermittent administration	Median follow-up duration was 24 months (range 9–120)	**Vit D 800 to 1000 IU/d + calcium:** **pooled RR 0.88 (95% CI 0.78–1.00) for osteoporotic fractures**

*RCT* randomized controlled trial, *25(OH)D* 25-hydroxyvitamin D, *Vit D* vitamin D, *RR* relative risk, *OR* odds ratio

Bold: statistically significant differences

In the ViDA trial in New Zealand, a monthly administration of 100,000 IU of vitamin D over about 3 years, after a loading dose of 200,000 IU, did not prevent fractures with even a non-significant trend for a higher fracture risk observed among healthy volunteers aged between 50 and 84 years and with a baseline 25(OH)D of 63 nmol/L [9]. Furthermore, an increase in falls and fractures was also previously observed with an annual high bolus of 500,000 IU of vitamin D in community-dwelling elderly Australian women at high risk of fracture, but with a baseline 25(OH)D considered as sufficient at around 50 nmol/L [50].

Many meta-analyses including various numbers of trials, hence of subjects, were recently published [47, 51–66] (Table 1). In that of Bolland et al. [59], which included 81 studies but with only 6% of vitamin D-deficient subjects at baseline, supplementation of vitamin D alone was not associated with a reduction of the incidence of total and hip fractures as well as of falls. Moreover this systematic review not only excluded trials combining vitamin D and calcium but included studies either with low doses of vitamin D or conversely high doses, especially bolus, which are known to increase falls and fractures [50, 67]. In another meta-analysis including 11 randomized trials enrolling at least 500 participants with the occurrence of at least 10 fractures representing more than 30,000 elderly subjects with baseline levels of 25(OH)D between 26 and 65 nmol/L, vitamin D supplementation alone did not decrease the risk of any or hip fracture [61]. In the meta-analysis of Yao et al. [61] involving 6 RCTs representing 49,282 participants including institutionalized subjects, the combination of vitamin D allowing a median increase of 23 nmol/L in 25(OH)D level and of 1000 to 1,200 mg/day of calcium supplementation, led to a 6% reduction in the risk of any fracture and a 16% reduction of hip fractures.

Regarding the effect of the combination of vitamin D and calcium on fracture prevention, a meta-analysis of 33 randomized trials involving more than 50,000 community-dwelling adults above 50 years of age and with an average baseline level of 25(OH)D at around 50 nmol/L did not show that vitamin D or calcium alone or the combination of vitamin D and calcium reduced the risk of fractures [57]. These negative results can be attributed to the exclusion of institutionalized subjects, to a very short follow-up for one-third of the studies and for the effect of vitamin D alone, two-third of the trials concerned vitamin D given as a bolus.

In a network meta-analysis which tested the effect of different doses of vitamin D, calcium and their combination in randomized controlled trials lasting more than one year in community-dwelling subjects aged over 50 years, vitamin D, calcium or both were not better than placebo or no treatment for the reduction of the risk of any or hip fractures [60]. Very recently, a systematic umbrella review of meta-analyses of controlled trials showed that the combined supplementation of vitamin D and calcium was associated with a reduction in the risk of hip fracture in two-third of 12 meta-analyses (RR 0.61–0.84) and a reduction in the incidence of any fracture in 7 of 11 meta-analyses (RR 0.74–0.95) with greater effect among institutionalized subjects [65]. It should be noted that the baseline levels of vitamin D available in 5 of these meta-analyses were between 21 and 83 nmol/L and that no fracture risk reduction was observed in meta-analyses exclusively evaluating community-dwelling individuals or in those on vitamin D alone [65]. Finally, the review of Li et al. [64] included not only pairwise meta-analysis of 35 trials, but also a Bayesian network meta-analysis. Oral vitamin D3 supplementation did not significantly reduce the risk of any fractures, but vitamin D3 alone at a dose of 700–800 IU/day and vitamin D3 plus calcium reduced by 9% and 15%, respectively, the incidence of any fractures in the pairwise meta-analysis. Similar results were obtained for hip fractures. However, no significant results were observed using Bayesian network meta-analyses [64]. Finally, in the most recent meta-analysis [66], 800–1000 IU/day of vitamin D and vitamin D together with calcium were associated with a 13% reduction in osteoporotic fracture risk.

In summary, intervention studies in elderly subjects with vitamin D deficiency, as demonstrated by low serum 25(OH)D, have shown a beneficial effect of vitamin D (800–1000 IU/day) and calcium supplementation on any and hip fractures. Vitamin D-deficient adults with 25(OH)D levels below 50 nmol/L could benefit from vitamin D and calcium supplementation that brings them into a 25(OH)D range of 50–100 nmol/L. On the other hand, vitamin D-replete adults with 25(OH)D levels in the range of 50–100 nmol/L are unlikely to benefit from vitamin D supplementation. Furthermore, vitamin D supplementation resulting in 25(OH)D levels above 100 nmol/L probably increases the risk of fractures*.*

### Vitamin D and falls

According to some falls prevention guidelines, vitamin D supplementation is one of the components of multifactorial interventions, together with strategies aimed at minimizing medications, initiating individually tailored exercise program, treating vision impairment, managing postural hypotension, heart rate and rhythm abnormalities, curing foot and footwear problems, and modifying the house environment [68, 69].

Vitamin D deficiency is associated with a higher risk of falls but vitamin D supplementation trials have produced discordant results, probably due to different doses and dosing intervals, calcium co-administration, observation duration, and disregard of 25(OH)D at baseline and follow-up. Indeed, many randomized controlled trials with vitamin D and calcium supplementation contain substantial, and sometimes fatal design flaws [70]. To be informative as well as to be included in a systematic review, a randomized controlled trial should include features such as: use of a single form of vitamin D and not either 1-a-hydroxyvitamin D or calcitriol as recently reviewed [71, 72]; use of a low exposure control group, and the intervention must be large enough to produce a meaningful change in nutrient status; adequacy of dose in the treatment group and compliance to the treatment; demonstration/documentation of the depleted status at baseline and adequate status with a “therapeutic” blood level during follow-up; use of a uniform response measure and optimization of co-nutrient status such as calcium intake [70]. Overall, baseline 25(OH)D level is a key issue as an inclusion criteria and a cut-off has still to be identified as well as the serum 25(OH)D to be reached to obtain the beneficial effect [73].

If the effect of vitamin D supplementation is considered in the general population, no fall prevention can be detected as reported in a Cochrane systematic review [74] Table 2. On the other hand, a highly significant decrease of 43% of the risk of falls was observed in the two studies with lower baseline levels of 25(OH)D [74]. This observation has been confirmed in another meta-analysis in which a 15% decrease in the risk of falls was obtained when 90% of the population had a baseline vitamin D level below 75 nmol/L; whereas, there was no preventive effect of vitamin D in the replete population [75] (Table 2).

**Table 2:** Meta-analyses of studies on vitamin D supplementation and falls

Author, year	Intervention	Target population, duration and baseline 25(OH)D	Outcome: falls
Bischoff-Ferrari et al. (2009) [92] 7 RCTs *N* = 1921	**Vit D ± calcium**	Individuals aged 65 years and older with a minimum follow-up of 3 months	**Pooled RR 0.81 (95% CI 0.71–0.92) with Vit D 700 to 1000 IU daily ± calcium**
			**Pooled RR 0.77 (95% CI 0.65–0.90) in those with achieved 25(OH)D ≥ 60 nmol/L**
Murad et al. (2011) [93] 26 RCTs *N* = 45,782	**Vit D ± calcium**	78% women, mean age 76 years, high risk of falling (15–69%, median 50%); duration: 3–62 months (median 12 months) Included both Vit D-deplete and -replete individuals, with info on Vit D status	**OR 0.86 (95% CI 0.71–0.92)**
			**Effect more prominent in vit D-deficient patients at baseline and in studies in which calcium was co-administered with vit D**
Gillepsie et al. (2012) [74]	**Vit D**	Most community-dwelling older people 7 RCTs; *N* = 9324	RR 1.00 (95% CI 0.90–1.11)—no reduction of the rate of falls
		13 RCTs; *N* = 26,747	RR 0.96 (95% CI 0.89–1.03)—no reduction of risk of falls
		In those with low vit D level at baseline: 2 RCTs; *N* = 260 3 RCTs; *N* = 562	**RR 0.57 (95% CI 0.37–0.89) for rate of falls** **RR 0.65 (95% CI 0.46–0.91) for number of fallers**
LeBlanc et al. (2015) [75] 11 RCTs *N* = 5682	**Vit D**	11 RCTs; *N* = 5682	**RR 0.89 (95% CI 0.82–0.97)**
		Low vit D levels (90% of population < 75 nmol/L): 7 RCTs; *N* = 2118	**RR 0.85 (95% CI 0.73–0.98) in bold**
		Not low vit D levels or not reported: 4 RCTs; *N* = 3564	**RR 0.89 (95% CI 0.83–1.04 not in bold since not significant**
Wu et al. (2017) [94] 26 RCTs *N* = 32,686	**Vit D** (ranged from 200 to 1000 IU/day in 11/26 RCTs dosage was 800 IU/day, 6 RCTs used a total dosage ranging from 300,000 IU/36 months to 600,000 IU/6 months) ** ± calcium**	Mean age ± SD of participants in these studies varied from 67 ± 2 to 92 ± 6 years Duration: 1–60 months	**OR for experiencing at least one fall, 0.87 (95% CI 0.80–0.94) with combined vit D + calcium**
Guirguis-Blake et al. (2018) [95] 7 RCTs *n* = 7531	**Vit D ± calcium** Vit D (700 IU or 800 IU daily, 150 000 IU every 3 months or 500 000 IU annually) 2 trials administered 1 μg of 1-hydroxycholecalciferol daily or 0.25 μg of calcitriol twice daily	Mean age: 71–77 years Vit D for 9 months up to 5 years Excluded studies on Vit D deplete Baseline mean serum 25(OH)D levels ranging from 65.9 to 79.4 nmol/L	Vit D did not prevent falls **Annual high-dose cholecalciferol (500,000 IU) showed an increase in falls, people experiencing a fall, and injurious falls; Calcitriol showed a reduction in falls and people experiencing a fall**
Bolland et al. (2018) [59] 37 RCTs *N* = 34,144	**Vit D alone in the majority of RCTs** with daily dose mostly < 1000 IU/day	Unselected populations of community-dwelling women aged 65 years or older	OR 0·97 (95% CI 0·93–1·02)
		Duration ≤ 1 year for the majority of studies	Results were similar in RCTs of high-dose versus low-dose vit D and in sub-group analyses of RCTs using doses greater than 800 IU per day
		Only 6% (4 studies) 25OHD < 25 nmol/L	**Comment by Bishoff-Ferrari (2019): considering 11 trials testing 800–1000 IU Vit D daily, with more than 50% adherence, and excluding large annual dosing trials, we see a significant falls’ reduction (RR = 0.88)**
		Non standardized lab measurements	
		Excluded trials with Vit D and calcium supplementation	
Thanapluetiwong et al. (2020) [63] 47 RCTs *N* = 58,424	**Vit D ± calcium** 37 trials with vit D3 7 trials with vit D2 1 trial with vit D2 and vit D3 2 trials with vit D analogues	Populations were mainly elderly women with age less than 80 years	**RR 0.948 (95% CI 0.914–0.984)** **By sub-group analyses, only Vit D with calcium supplement significantly reduce fall incidence, RR 0.881 (95% CI 0.821–0.945)** **Vit D3 supplement decreased incidence of fall but this occurred only when vit D3 was combined to calcium**
Ling et al. (2021) [96] 31 RCTs *N* = 57,867	**Vit D alone, daily or intermittent doses of 400–60 000 IU** 21 RCTs, *n* = 51,984	Only trials enrolling adults (age ≥ 18)	RR 1.00 (95% CI 0.95–1.05) **Subgroup analyses showed that with baseline of serum 25OHD < 50 nmol/L fall risk was reduced RR 0.77 (95% CI 0.61–0.98)**
	**Vit D (daily doses of 700–1000 IU) plus calcium (daily doses of 1000–1200 mg)** 10 RCTs, *n* = 5883		**RR 0.88 (95% CI 0.80–0.97)**
Kong et al. (2022) [66] 32 RCTs *N* = 104,363	**Median daily dose of Vit D: 800 IU**	Most studies included women (75% of participant; range 15–100%)	**Pooled RR 0.91 (95% CI 0.85–0.98) with daily Vit D dose of 800–1000 IU**
	21 RCTs, *N* = 36,793 with falls as outcomes		No reduction in studies with < 800 or > 1000 IU/day
	8 studies: < 800 IU/day	Median age was 72 years (range 53–85)	
	15 studies: 800–1000 IU/day, and	Median follow-up duration: 24 months (range 9–120)	**Daily administration of Vit D associated with reduced risk of falls, while intermittent dose was not**
	9 studies: > 1000 IU/day		
	26 studies reported daily administration, while 6 reported intermittent administration		**Patients with vit D deficiency showed a significant risk reduction of falls with Vit D supplementation**

*RCT* randomized controlled trial, *25(OH)D* 25-hydroxyvitamin D, *Vit D* vitamin D, *RR* relative risk, *OR* odds ratio

Bold: statistically significant differences

Regarding randomized controlled trials assessing the effect of vitamin D on falls, several studies showed a significant decrease of the risk of falls in participants who are vitamin D depleted at baseline and/or reached appropriate serum levels of 25(OH)D during the intervention [76–80]**.** There are also some interesting data from the DEX Trial showing no differences in fall risk in 409 home-dwelling women over 70 years of age with baseline vitamin D levels superior to 65 nmol/L [81–83], but when the same population was stratified for the levels of serum 25(OH)D reached during the 2-year follow-up, the highest quartile was associated with an about 37% lower risk of falls as compared to the lowest quartile[84]. Vitamin D supplementation doses higher than 1000 IU/day among community-dwelling older adults with an elevated fall risk and baseline 25(OH)D of 25 to 72.5 nmol/L might have differential effects on fall risk, with an increased risk of first time fall with fracture, but a decreased risk of outdoor falls [85].

Many randomized controlled trials have also not shown any reduction of falls with vitamin D supplementation. This lack of reduction in falls was probably either due to the populations studied which were already vitamin D replete at baseline [10, 86–89] or when high doses of vitamin D supplementation were administered [9, 90, 91].

Systematic reviews confirm that the reduction of falls with vitamin D supplementation is essentially observed in vitamin D deplete individuals [59, 63, 66, 74, 75, 92–96] (Table 2). Indeed, in the review by Bolland et al. [59] which concluded that vitamin D did not prevent falls based on 81 trials, 44 of these trials were with daily doses less than 1000 IU and only 6% of participants had baseline 25(OH)D below 25 nmol/L. A comment by Bischoff-Ferrari et al. [97] reported that by considering only the 11 trials testing 800–1000 IU daily with more than 50% adherence and excluding large annual dosing trials (which have been shown to be associated with higher rates of falls), a significant fall reduction of 12% was observed. Another systematic review including 7 trials on vitamin D supplementation focused on community-dwelling vitamin D-replete populations showed no prevention of falls [95]. More recent megatrials suggested that vitamin D supplementation in vitamin D-replete individuals does not provide any benefit and it was also clearly indicated that large bolus doses of vitamin D increased the risk of falls [98, 99]. Indeed there is evidence that high-dose vitamin D stimulates release of FGF-23 from osteocytes which impairs the 1-alpha hydroxylation of 25(OH)D to 1,25(OH)2D and also promotes 24-hydroxylation of 25(OH)D to the inactive form of 24, 25(OH)2D, and therefore excessive vitamin D may cause insufficiency of the active metabolite 1,25(OH)2D [100–102]

In summary, vitamin D3 supplementation with doses less than 800 IU/day does not seem to reduce falls while doses between 800 and 1000 IU/day reduce falls and large bolus doses increase falls. In vitamin D deplete patients and as part of a multicomponent intervention, vitamin D supplementation may be effective in reducing fall risk. Unfortunately, the recent 2017–2020 megatrials did not address the question of vitamin D and falls in vitamin D deplete populations.

### Vitamin D and osteoarthritis

The older adult population at risk of osteoporosis and falls is also at risk of osteoarthritis (OA). The effect of vitamin D on OA is unclear at least on the epidemiological point of view, but also at the intervention level, and may differ according to the severity of OA. The relationship between vitamin D and OA during OA development and progression is still debated. Observational studies have not shown any relationship between vitamin D status and OA development in patients with mean 25(OH)D above 50 nmol/L for pain, radiologic OA and cartilage volume loss [103]. A recent meta-analysis taking into account a series of confounders did not show significant associations between serum levels of 25(OH)D and the prevalence, incidence or progression of knee radiographic OA and joint space narrowing [104]. However, a sub-group analysis showed a significant associations between low vitamin D levels and progression of knee OA. A recent Mendelian randomization study involving 455,221 participants and taking into account the role of genetic background, indicated an inverse causal relationship between serum PTH concentrations and development of OA [105]. Moreover, a site-specific association was also observed between serum PTH levels and knee OA. The potential mechanisms by which serum PTH affects OA need to be further investigated. However, there was no evidence of a causal effect of serum 25(OH)D levels on OA [105]. In a large cross-sectional Italian study involving 2756 men and women with a mean age of 74.2 years, the relationship between 25(OH)D levels and any presence of OA and pain was examined [106]. Considering the subjects in the highest 25(OH)D quartile as a reference, those in the lowest quartile had significantly higher odds of OA and OA-related pain, particularly when the hand and hip were involved.

Randomized controlled trials on the effects of vitamin D supplementation on the progression of knee OA and related symptoms were recently summarized in several meta-analysis [103, 107–111] and are presented in Table 3 including various studies [112–118]. Vitamin D supplementation to patients with 25(OH)D below 50 nmol/L, but not higher, may alleviate pain and improve joint function. In the review of Mathieu et al. [111], 3 studies assessing the effects of 2000 to 3000 IU of vitamin D per day for 1 or 2 years on OA symptoms in more than 500 patients demonstrated that VAS evaluated pain and WOMAC function were significantly improved, albeit with a modest effect size.

**Table 3:** Meta-analyses of studies on vitamin D supplementation and knee osteoarthritis

Author	RCT number and sample size	Vit D intervention	Outcome
Gao et al. (2017) [107]	4 RCTs; *n* = 1136	800–2000 IU daily or 50,000–60,000 IU monthly	**↓ WOMAC pain: score − 1.65 (95% CI − 2.16 to − 1.14) with 2000 IU/day**
			**↑ WOMAC function: score -1.87 (95% CI − 2.58 to − 1.17) with 2000 IU/day**
			No effect on **WOMAC** stiffness and tibia cartilage volume
Diao et al. (2017) [108]	4 RCTs; *n* = 1136	800–2000 IU daily or 50,000–60,000 IU monthly	**↓ WOMAC pain: SDM -0.32 (95% CI − 0.63 to − 0.02)**
			No effect on tibia cartilage volume or joint space width, regardless of baseline 25(OH)D levels
Beaudart et al. (2020) [109]	4 RCTs; *n* = 1136	800–2000 IU daily or 50,000–60,000 IU monthly	**↓ WOMAC pain: SDM -0.31 (95% CI − 0.56 to − 0.06)**
			**↑ WOMAC function: SDM -0.30 (95% CI − 0.49 to − 0.11)**
Zhao et al. (2021) [110]	6 RCTs; *n* = 1599	800–2000 IU daily or 50,000–60,000 IU monthly	**↓ WOMAC pain: SDM -0.32 (95% CI − 0.63 to − 0.02)**
			**↑ WOMAC function: SDM -0.34 (95% CI − 0.60 to − 0.08)**
			**↑ WOMAC stiffness: SDM -0.13 (95% CI − 0.26 to − 0.01)**
			No effect on tibia cartilage volume or joint space width
Mathieu et al. (2022) [111]	3 RCTs; *n* = 662	2000 IU daily or 50,000–60,000 IU monthly	**↓ WOMAC pain: SDM -0.20 (95% CI − 0.35 to − 0.04)**
			**↑ WOMAC function: SDM − 0.44 (95% CI − 0.80 to − 0.09)**

Bold value indicates statistically significant differences

*Vit D* vitamin D, *RCT* randomized controlled trial, *SDM* standardized mean difference

In summary, observational and intervention studies provide little evidence for a protective effect of vitamin D on cartilage volume loss or radiologic OA worsening, although it may have a favorable effect on joint pain. Indeed, subset analyses and one pilot randomized controlled trial suggest that patients with 25(OH)D below 50 nmol/L may experience less joint pain with vitamin D supplementation. Trials assessing radiologic OA and cartilage loss did not demonstrate an effect of vitamin D supplementation in patients with 25(OH)D levels above 50 nmol/L. No trials have been performed in patients with low 25(OH)D levels.

### Metabolic conditions and treatment with calcifediol (25-hydroxyvitamin D (25(OH)D))

The synthesis of calcifediol depends on the individual synthetic potential of CYP2R1, CYP24A1, CYP3A4, CYP2D25 enzymes [119, 120]. The mechanisms of control of CYP2R1 are unknown, even if there are some data on the effects of calcitriol, phenobarbital, glucocorticoids and antiretroviral drugs. Calcifediol administration produces rapid (within hours) increases in plasma 25(OH)D levels [121]. Calcifediol given daily, weekly, or as a single bolus is about two to three times more potent and more rapid in increasing plasma 25(OH)D concentrations than vitamin D in a double-blind randomized controlled trial in 35 Caucasian postmenopausal women aged between 50 and 70 years of age [122]. In another randomized controlled trial, 20 healthy postmenopausal women with an average 25(OH)D level of 33 nmol/L (13.2 ng/ml) and a mean age of 61.5 years were randomized to 20 μg of calcifediol or to 20 μg (800 IU) of vitamin D3 daily [123]. At 4 months, mean 25(OH)D levels increased to 173 nmol/L (69.3 ng/ml) in the calcifediol group and to 76 nmol/L (30.5 ng/ml) in the vitamin D group (p < 0.0001). Calcifediol improved gait speed by 18% compared with vitamin D [123]. In another randomized controlled trial, 303 postmenopausal women with baseline levels of serum 25(OH)D below 50 nmol/L (20 ng/ml) were randomized 1:1:1 to calcifediol 0.266 mg/month for 12 months, calcifediol 0.266 mg/month for 4 months followed by placebo for 8 months, and cholecalciferol 25,000 IU/month for 12 months [124]. At month 4, 35% of postmenopausal women treated with calcifediol and 8.2% of those treated with cholecalciferol reached serum 25(OH)D levels above 75 nmol/L (30 ng/ml) (*p* < 0.0001). This difference was already observed after the first month of treatment and confirms that calcifediol is effective, faster, and more potent than cholecalciferol in increasing serum 25(OH)D levels [124].

Calcifediol may be an option for managing vitamin D deficiency in obese or malabsorptive patients who have difficulty increasing serum 25(OH)D with vitamin D supplementation. Indeed, in a pharmacokinetic study of a randomized, double-blind crossover trial, AUCs of 900 ug calcifediol were not significantly different between either malabsorptive patients and healthy participants, and between participants with higher BMI and those with lower BMI whereas AUCs of 900 ug vitamin D3 were significantly lower by 64% and 53%, respectively[102].

Calcifediol in an extended-release formulation (ERC) could be a novel approach to manage secondary hyperparathyroidism linked to 25(OH)D insufficiency in non-dialysis CKD [125]. Indeed, ERC has been shown to increase 25(OH)D gradually and provide a physiologically regulated increase in 1,25(OH)2D that can reliably lower PTH in CKD stage G3–G4 without clinically meaningful increases in serum calcium and phosphate levels [126]. Patients with nephrotic syndrome have low blood levels of 25(OH)D. Oral therapy with 200 ug calcifediol normalized plasma levels of 25(OH)D within 48 h in patients with nephrotic syndrome though intestinal calcifediol absorption was delayed and its elimination rate was enhanced as compared to control subjects [127].

In men with hypogonadism characterized by low levels of 25(OH)D, supplementation with calcifediol (4000 IU or 100 ug per week) for 3 months, but not the administration of cholecalciferol (5000 IU per week), increased 25(OH)D and decreased PTH levels [128]. Among renal transplant recipients, insufficient or deficient 25(OH)D levels are highly prevalent, and these deficits improved with moderate doses of oral calcifediol without side effects [129].

Another condition in which a rapid correction of inadequate vitamin D levels has been advocated is COVID-19. While the available evidence based largely on poor-quality observational studies may show a trend for an association between low serum 25(OH)D levels and COVID-19 related health outcomes, this relationship was not found to be always statistically significant [130–132]. Calcifediol supplementation may have a protective effect on COVID-19-related ICU admissions in an observational cohort study [133] and in a pilot randomized clinical study [134] and was also associated with lower in-hospital mortality during the first 30 days [135] as well as a lower mortality in patients achieving serum 25(OH)D levels ≥ 75 nmol/L [136], as compared with those not receiving calcifediol. Even moderate doses of 5000 IU daily for 2 weeks was associated with faster resolution of COVID-19 symptoms [137]. The current use of high doses of vitamin D in COVID-19 patients is not based on solid evidence. Very recently in a randomized controlled trial with 50 subjects hospitalized for COVID-19 with a mean age of 65 years and a baseline 25(OH)D level at 55 nmol/L, 25,000 IU cholecalciferol/day over 4 consecutive days followed by 25,000 IU/week up to 6 weeks reduced the hospitalization duration (4 days vs 8 days) and the need for supplemental oxygen [138].

In summary, the administration of calcifediol is superior to cholecalciferol in conditions requiring a rapid increase in 25(OH)D and in conditions of liver insufficiency (Table 4, adapted from [139]).

**Table 4:** Conditions in which the administration of calcifediol may be preferable to cholecalciferol

Inactivating mutations of genes encoding hepatic 25-hydroxylase
Iatrogenic inhibition of hepatic 25-hydroxylase [e.g., anticonvulsants, antiretroviral drugs, glucocorticoids and bisphenol A]
Hepatic insufficiency
Decreased bioavailability (adipose tissue sequestration)
Fat malabsorption
CKD—renal osteodystrophy
Nephrotic syndrome (proteinuria)
Male hypogonadism
Other conditions (e.g., diabetes mellitus I, transplant recipients)
Long-lasting osteomalacia
COVID-19

### Vitamin D safety and therapeutic window

The upper limit of safety for vitamin D intake is suggested to be 4000 IU/day according to the Institute of Medicine [45]. For serum total 25(OH)D levels, the Institute of Medicine and the US Endocrine Society used different cut-points to define deficiency, insufficiency and sufficiency as well as for a threshold of possible harm (125 and 250 nmol/L, respectively) [140]. Issues in vitamin D supplementation safety concern the dose of vitamin D, the outcomes such as serum levels and urinary excretion of calcium, falls and bone, the schedule of administration and the target population (e.g., Vitamin D replete vs deficient, obese, comorbidities, etc.) [141]. In vitamin D intoxication, there is persistence of abnormally elevated fasting urinary calcium and of serum 25(OH)D concentrations, long after normalization of plasma calcium, indicating that calcemia may be not sensitive enough to detect vitamin D overdosing [142]. A dose–response relationship based on randomized controlled trials’ results was observed between higher dose and higher achieved 25(OH)D serum values and fall and fracture prevention with optimal benefits with 700 IU–1000 IU vitamin D3/day or mean serum 25(OH)D levels between 75 and 110 nmol/L [143]. In a 12-month double-blind randomized controlled trial, elderly women with a mean age of 66 years and a relatively low baseline 25(OH)D of 38 nmol/L were randomized to one of seven daily oral doses of vitamin D3 (range 400–4800 IU) or placebo [144]. The maximum decrease in falls was obtained with serum 25(OH)D of 75–100 nmol/L and median doses 1600–3200 IU while fall rates increase as serum 25(OH)D exceeded 100 to 112.5 nmol/L suggesting a biphasic effect (U-shape curve) of vitamin D effect [144]. Likewise, in healthy community-dwelling postmenopausal women with vitamin D insufficiency (25(OH)D < 50 nmol/L), a relatively high dose of vitamin D3 (2800 IU/day) had no beneficial effects on muscle strength and physical performance with even some deleterious effect on handgrip and knee flexion 60° [145]. Furthermore, in a one-year double blind randomized controlled trial, community-dwelling men and women aged 70 years and older amongst whom 58% were vitamin D insufficient (< 50 nmol/L), received 24,000 IU vs 60,000 IU vitamin D3 vs 24,000 IU vitamin D3 and 300 ug calcifediol [146]. Although higher monthly doses of vitamin D were effective in reaching a 25(OH)D threshold of at least 75 nmol/L, participants had no benefit on lower extremity function, but an increased risk of falls was even observed in the highest quartile of achieved 25(OH)D of 125 nmol/L as compared to the lowest quartile with 25(OH)D at 67 nmol/L [146]. Finally, in the Australian D-Health trial, a monthly dose of 60,000 IU of cholecalciferol given to 21,315 participants aged 60–84 years for a maximum of 5 years with mean achieved serum 25(OH)D concentrations of 114.8 nmol/L did not reduce the risk of falling, but even was associated with a higher risk in a sub-group with BMI < 25 kg/m^2^ [91].

The importance of the outcome and of the target population in evaluating vitamin D dosing and efficacy was well illustrated in a randomized controlled trial among older long-term care residents in whom high-dose vitamin D3 supplementation of 3000–4000 compared to 400–1000 IU/day for 12 months reduced the incidence of acute respiratory infection but increased the rate of falls [147]. Another prospective observational study in older community-dwelling men followed for 4.3 years highlighted a safe target range for serum 25(OH)D concentrations since the risk for fracture was greatest in men with 25(OH)D levels in the lowest quintile (< 36 nmol/L) as well as in men in the highest quintile (> 72 nmol/L) [46]. Regarding bone microstructure, vitamin D given for 3 years at a dose of 4000 IU or 10,000 IU compared with 400 IU/day in healthy men and women with a mean age of 62 years and a baseline 25(OH)D level of 79 nmol/L resulted in statistically significant lower volumetric bone density at the distal radius and tibia with the 10,000 IU/day dose [148]. These deleterious effects of high-dose vitamin D supplementation with 4000 or 10,000 IU, compared with 400 IU daily, resulted in greater losses of total volumetric BMD in healthy vitamin D-sufficient females, but not in males [149].

Regarding intermittent regimens of vitamin D supplementation, similar serum 25(OH)D concentration were achieved by 2 months with vitamin D3 supplementation of 1500 IU daily, 10,500 IU once weekly, or 45,000 IU once monthly in elderly women randomized after hip fracture surgery [150]. In some European countries, 25,000 IU monthly (corresponding thereby to 800 IU/day) is commonly prescribed. There is no evidence to recommend or to discourage this regimen. The choice may be left to the patient’s preference for ensuring optimal adherence to vitamin D supplementation.

In a large randomized controlled trial in UK among community-dwelling men and women, 100,000 IU oral vitamin D3 given every four month over five years, corresponding to about 800 IU daily, prevented fractures in this non-depleted vitamin D population with serum 25(OH)D measured in a small sub-group of 74.3 vs 53.4 nmol/L in the placebo group after four years [151]. On the other hand, among older community-dwelling women considered to be at high risk of fracture, annual oral dose of 500,000 IU of cholecalciferol for three years resulted in an increased risk of falls and fractures which was already observed within 3 months after dosing [50].

Is a vitamin D loading dose useful and/or safe? In a randomized controlled trial in vitamin D, non-depleted elderly participants with baseline 25(OH)D of 58 nmol/L, a loading dose of 500,000 IU showed a rapid increase in 25(OH)D up to 120 nmol/L by one month and then reached a plateau of about 80 nmol/ by 3–5 months whereas it took 3–5 months to obtain a similar plateau with 50,000 IU monthly of vitamin D supplementation [152]. A loading dose of 600,000 IU in elderly subjects increased sCTX already by day 1, which remained sustained for two months, whereas sCTX changes with 300,000 and 100,000 IU were considerably lower and reached statistical significance only within the first 3 days with the 300,000 IU dose [153]. Finally, a single bolus dose equivalent to 700 IU/day of vitamin D3 supplementation in postmenopausal women with a mean age of 65 years, did not improve distal radius fracture healing over a 6 week observation while a bolus equivalent to 1800 IU/day may be detrimental in restoring bone stiffness during the first 12 weeks of fracture healing [154]. These results call for some caution in using large loading doses [155].

In summary, daily doses of 800–1000 IU of vitamin D are safe, while intermittent regimens with doses higher than those equivalent to this daily dosing are not recommended. The risk of overdosing expression is related to the outcome evaluated. The upper limit of safety for clinical outcomes (both for dose and circulating levels) should be better evaluated and defined.

## Conclusion

Considering the well-recognized major musculoskeletal disorders associated with severe vitamin D deficiency and taking into account a possible biphasic effect of vitamin D, the results of various meta-analyses of randomized controlled trials on the effects of vitamin D on fracture risk, falls or osteoarthritis, indicate that 1000 IU daily should be recommended in patients at increased risk of vitamin D deficiency (Table 5). This regimen is safe. Some groups of patients may benefit from a vitamin D loading dose or from calcifediol treatment to early achieve 25-hydroxyvitamin D therapeutic levels.

**Table 5:** Indications for vitamin D supplementation

Daily vitamin D (800–1000 IU)
Subjects at risk of osteoporosis
Patients on concurrent osteoporosis treatment
Patients with fragility fracture
Elderly people at risk of falling
Obese patients
Subjects with pigmented skin
Subjects with limited sun exposure
Subjects with insufficient vitamin D intake
Patients with malabsorption^a^
Patients after bariatric surgery^a^
Patients on anticonvulsants
Patients on glucocorticoids
Loading dose (25,000 or 50,000 IU/week for 4–6 weeks)
Low 25-hydroxyvitamin D levels
Need for a rapid correction of vitamin D deficiency
After bariatric surgery
Malabsorption
Severe obesity

In some countries, 25,000 IU monthly (corresponding thereby to 800 IU/day) is commonly prescribed, and might be acceptable if it meets patients´ preference despite equivalence with the daily dose was not clearly established

^a^Higher doses (2000 IU/daily) may be needed
